# Friend or foe? Concentration of a commensal microbe induces distinct responses in developing honey bees exposed to field-realistic pesticide concentrations

**DOI:** 10.1093/femsec/fiaf080

**Published:** 2025-07-29

**Authors:** Monika Yordanova, Xiao Zhang, Carlota B Torres, Sophie E F Evison, Richard J Gill, Peter Graystock

**Affiliations:** Georgina Mace Centre for the Living Planet, Department of Life Sciences, Imperial College London, Silwood Park Campus, Ascot SL5 7PY, United Kingdom; Department of Biology, University of Oxford, Wellington Square, Oxford OX1 3SZ, United Kingdom; Georgina Mace Centre for the Living Planet, Department of Life Sciences, Imperial College London, Silwood Park Campus, Ascot SL5 7PY, United Kingdom; Department of Biology, University of Oxford, Wellington Square, Oxford OX1 3SZ, United Kingdom; Georgina Mace Centre for the Living Planet, Department of Life Sciences, Imperial College London, Silwood Park Campus, Ascot SL5 7PY, United Kingdom; Division of Evolution & Genomics, School of Biological Sciences, University of Manchester, Manchester M13 9PL, United Kingdom; Georgina Mace Centre for the Living Planet, Department of Life Sciences, Imperial College London, Silwood Park Campus, Ascot SL5 7PY, United Kingdom; Georgina Mace Centre for the Living Planet, Department of Life Sciences, Imperial College London, Silwood Park Campus, Ascot SL5 7PY, United Kingdom

**Keywords:** commensalism, *Enterococcus*, honey bee, larvae, pesticides

## Abstract

Commensal microbes play important roles in modulating host health through varied mechanisms. *Enterococcus faecalis*, a Gram-positive commensal bacterium found across a wide range of hosts, has the potential to benefit its host through probiotic, antimicrobial and detoxification properties. However, it can also cause adverse effects, disrupting the host's healthy microbial communities and responses to co-stressors. Its context-dependent impact on the health of the agriculturally important pollinator - *Apis mellifera -* has been sparsely explored. Here, we examined the effects on honey bee brood survivorship and development when exposed at different concentrations and when co-exposed with chemical stressors (acetamiprid, thymol, glyphosate, and a mixture of the three). We found high doses of *E. faecalis* significantly reduced larval survivorship and size of brood at multiple developmental stages. Conversely, we found that low doses of *E. faecalis* increased larval size when individuals were co-exposed to the pesticide mixture. We also found that glyphosate alone and the pesticide mixture reduced the mass of brown-eyed pupae. These results are the first to show the dual role of *E. faecalis* in honey bee health is dependent on the concentration of the microbe and the co-stressors that brood are exposed to.

## Teaser

The direction and magnitude of impact *Enterococcus faecalis* has on honey bee host health is influenced by dose and co-exposure to pesticide.

## Introduction

Commensalism has traditionally been defined as an inconsequential relationship (Zinsser and Bayne-Jones 1939). However, commensal microbes have the potential to incur divergent impacts influencing host health depending on the context. Whilst serving as beneficial guardians of homeostasis, the same microbe species may also be involved in pathogenic processes, either as a pathogen themselves or through facilitating pathogenesis (Hayes et al. 2018, Dey and Ray Chaudhuri 2023). Parasitism and mutualism, envisioned as opposite outcomes on the symbiotic spectrum, are not fixed labels of a species function but rather a context-dependent description of the host–microbe interaction at a given moment or environment (Bordenstein et al. 2009). An example of this is the common mammalian gut microbe*-Escherichia coli* (Kaper et al. 2004, Sewell et al. 2018, Eberl et al. 2021). Immunocompromised hosts may be more susceptible to high concentrations of *E. coli* as this stimulates the production of host autoinducer signals, triggering *E. coli* to transition to a pathogenic state (Kaper et al. 2004). Furthermore, exposure to additional stressors can amplify the negative effect of *E. coli*. For instance, chemicals such as antibiotics can disrupt the host's microbial community and allow pathogenic *E. coli* to proliferate and further damage the host (Kaper et al. 2004). Yet, under different conditions, *E. coli* can also aid its host in utilising important nutrients (Sewell et al. 2018) and reduce infections with other intestinal pathogens (Guo et al. 2019, Eberl et al. 2021). Similar divergent effects on the host under different contexts have been reported in the intracellular bacteria *Wolbachia* (Bi and Wang 2020). *Wolbachia* is a common intracellular host pathogen which employs various strategies such as parthenogenesis, feminisation, male killing, and cytoplasmic incompatibility to manipulate host reproduction, ensuring its own successful proliferation and transmission at a significant cost to its host (Bi and Wang 2020). Outside of reproductive tissues, the pathogen can also have consequences to host learning, memory and circadian rhythms (Bi and Wang 2020). Despite this, it can beneficially manipulate host microbiota, promoting the growth of microbes which aid the host in coping with chemical stressors (Ourry et al. 2021). In some cases, it has become so integral to some of its hosts, it has become necessary for oogenesis and reproduction (Pannebakker et al. 2006). Further, it can interact with its host, promoting increased immune responses against pathogens (Zhang et al. 2020). These examples allude to the complex context-dependent function of bacteria and divergent interactions with their host, which may otherwise be overlooked when broadly labelling as pathogens, commensals, or useful symbionts.

**Figure 1.**
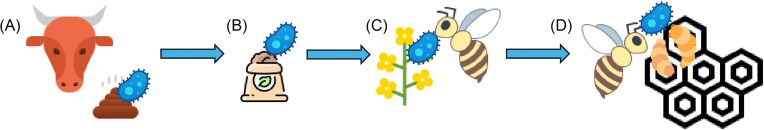
Exposure of honey bee larvae to *E. faecalis*. The bacterium is represented using blue bacterial icon. A) It is a commensal microbe found in a broad range of species. B) The excrement of some of these species can act as a nutrient-rich fertilizer, which may contain the bacterium. C) As the fertilizer is used on crops, bees can become exposed to *E. faecalis* through feeding on the pollen and nectar of these crops. D) Adult bees can introduce *E. faecalis* to larvae in their hives by contaminating their food (31–37). Icons by Icon8.com

The bacteria in the genera *Enterococcus* have been reported to show a diversity of effects in a wide range of hosts (Krawczyk et al. 2021). *Enterococcus faecalis* is a ubiquitous Gram-positive bacterium commonly encountered in soil, water, plants, and animals (Mundt 1963, Müller et al. 2001, Sánchez Valenzuela et al. 2012, Daniel et al. 2017, Weiss et al. 2017). Yet, despite its prevalence, work examining context-dependent effects within the same host is limited. Here, we look to determine what factors can cause such divergent responses and do this by examining the effects on the development and survival of a commercially important host-the western honey bee, *Apis mellifera* (Hung et al. 2018, Hristov et al. 2020, Reilly et al. 2020). Adult honey bees likely encounter *E. faecalis* whilst foraging on contaminated flowers, which have been shown to be hubs of parasite transmission (Graystock et al. 2015, Graystock et al. 2020, Sánchez Valenzuela et al. 2012). The bacterium is more common in nectivorous insects (compared to stem or leaf piercing ones), and it has been found within a wide range of bee species (Martinson et al. 2012, Parichehreh 2017, Dickel et al. 2018, Praet et al. 2018). Within honey bees, *E. faecalis* is considered to have neutral effects (Lewkowski and Erler 2019). Adult bees can bring *E. faecalis* contaminated floral nectar and pollen into their hives and incorporate it into the bee bread, which brood feed on (Fig. 1) (Sánchez Valenzuela et al. 2012, Mohammad et al. 2020, Lanh et al. 2022). The bacterium is especially common in colonies infected with European foulbrood (a destructive disease of honey bee larvae caused by the bacterium *Melissococcus plutonius*) and is considered a secondary invader for the infection (Anderson et al. 2023, Erban et al. 2017a). Despite this association, it is hypothesized that *E. faecalis* can have positive effects on honey bees due to its antimicrobial effects *in vitro* (Jaouani et al. 2014) and ability to increase survivorship of individuals infected with disease such as *Nosema* (Borges et al. 2021). Considering the variable effects of *E. faecalis* on the host and the wide range of stressors honey bees are increasingly facing, it is important to understand the context-dependent role of this common commensal microbe to better understand the risks (or benefits) bees face.

Honey bees encounter a variety of stressors to their health and productivity, including pesticide exposure and disease, which can interact to further diminish the host’s health (Siviter et al. 2021, Yordanova et al. 2022). Honey bees lay eggs that develop into larvae, then pupae, before eclosing as an adult bee. This developmental ‘brood’ stage of pre-adult bees is immobile and especially vulnerable to parasite stress due to their non-specialised immune systems (Laughton et al. 2011). Honey bee larvae are fed by young bees (nurse bees) and can become exposed to a cocktail of stressors such as pesticides and microbial pathogens whilst being fed (McKee et al. 2003, Mullin et al. 2010). Pesticides can have sublethal effects on larval health, including delayed development or reduced size, which can impede overall colony productivity (Bryden et al. 2013). Pesticide stress can also alter host–microbe interactions by manipulating immunity, therefore amplifying the risk of pathogenesis (Di Prisco et al. 2013, Pamminger et al. 2018, Yordanova et al. 2022). In addition, the effects of toxicogenic bacteria on larvae can be heightened in the presence of additional toxic compounds, as has been shown for the *Paenibacillus larvae* parasite in combination with the pesticides dimethoate (organophosphate) and clothianidin (neonicotinoid) (López et al. 2017). Despite this, thiacloprid (neonicotinoid) and *Enterococcus faecalis* co-exposure increases the survivorship of adult honey bees, showing its effects on honey bees may also be positive (Dickel et al. 2018). No studies have previously examined the interactive effects between a commensal microbe’s dose and pesticides on honey bee brood development.

In this study, we examined whether different levels of *E. faecalis* exposure influence the survival and growth of different stages of honey bee brood development and whether there is an interaction between this and co-exposure to a number of pesticides. We anticipated that *E. faecalis* dose may play a role in larval survival and growth, which may be influenced by our tested pesticides and potentially lead to divergent effects on the host.

## Materials and methods

### Experimental bees

We obtained larvae from two hives maintained at the Observatory Ridge apiary in Silwood Park (Berkshire, England). Each colony had no known prior exposure to pesticides or visible signs of pests and diseases. We reviewed the brood frames the day prior to the experiment to mark frames containing eggs. We extracted the brood frames in May 2023 with the aim of minimising heat loss and maintaining humidity. The air temperature was >15°C and wind <24mph, with each extracted frame covered with a warm damp towel and placed in high-density polystyrene nucleus lined with heat packs maintained at 34–36°C until transported to a 35°C incubator (Oecd 2016).

We grafted 720 healthy first-stage larvae (L1, no older than 24 h) from the brood frames into sterile 48-well tissue plates, with each well containing 30μl of pre-prepared larval feed (Oecd 2016) (Supporting materials) containing the respective pesticide treatments or controls. We incubated the larvae at 35±0.5°C, and humidity 95±5% RH maintained using a saturated K_2_SO_4_ solution (Crailsheim et al. 2013, Schmehl et al. 2016). Larval feed was prepared according to Schmehl Diet C (Schmehl et al. 2016) and selected based on survivorship outcomes of a preliminary trial (Supporting materials).

### Cultivation of *E. faecalis*

We isolated *E. faecalis* from six larval samples with symptomatic European Foul Brood disease that were collected by the National Bee Unit inspectors in 2021 (NBU). To achieve this, we grew mixed cultures from larval sample homogenates in basal media (BA) under anaerobic conditions at 35°C. The basal media per 1 l consisted of 20 g agar, 10 g of soluble starch, 10 g of yeast extract, 10 g of glucose, 1 g. L-cysteine, and approximately 100 ml of 1 M KH_2_PO_4_ (until a pH of 6.7 was reached). We then sub-cultured colony-forming units (CFUs) based on morphology and transferred them to liquid broth, where they were incubated overnight and stored in 15% glycerol at −80°C for future experimentation (Zalomova et al. 2021). In addition, a subsample of each isolate had its DNA extracted using a Qiagen DNEasy Blood & Tissue kit (DNeasy Blood and Tissue Handbook 2020). DNA was screened for *E. faecalis* using Polymerase Chain Reaction (PCR) with *Enterococcus* primers atpA-20-F (TAYRTYGGKGAYGGDATYGC) and atpA-27-R (CCRCGRTTHARYTTHGCYTG), followed by Sanger sequencing at Eurofins to confirm identity (Naser et al. 2005).

### Selection of pesticides

We selected three toxic compounds (acetamiprid, glyphosate, and thymol) that we know honey bees can be exposed to during husbandry and/or across landscapes, and based on known applications to crops visited by bees (Manzano Sánchez et al. 2021, Ridley et al. 2021). Acetamiprid is a neonicotinoid insecticide used worldwide (Fischer et al. 2014, Liu et al. 2024) and has been found inside honey bee hives (Pohorecka et al. 2017, Böhme et al. 2018, Daniele et al. 2018, Bokšová et al. 2021, Stehle et al. 2023). Glyphosate is the most highly used herbicide in the United States and Europe (Benbrook 2016, Álvarez et al. 2023, Ridley et al. 2023). It is commonly detected at high concentrations in European honey bee hives (El Agrebi et al. 2020, Bergero et al. 2021). The most common chemicals often detected in colonies are miticides, which can negatively impact honey bee health and development (Mullin et al. 2010, Glavan et al. 2020, Qi et al. 2020). As concerns surrounding miticides mount, many countries have encouraged beekeepers to swap the commonly used chemical formulations for ‘natural’ alternatives such as thymol (Committee on Agriculture and Rural Development 2018).

We exposed larvae to chemical concentrations detected in the field (Table 1, further details in Supporting materials). In addition to treatments exposing bees to a single chemical, we had a treatment with a mixture of all three (named trimix) to mimic realistic conditions since it is known that honey bee larvae are frequently exposed to a mixture of pesticides (Mullin et al. 2010). Additionally, to confirm our experimental setup was indeed exposing bees to the chemical treatments, we included a positive chemical control, dimethoate, which is known to have lethal effects on larvae when applied at the OECD recommended concentration 48 mg/kg (Oecd 2016). We diluted all pesticides to the desired concentration using water; hence, we added water to the larvae's feed in the negative control group to ensure the same volume was displaced across treatments.

**Table 1.** Pesticides used in the experiment, the concentrations they have been encountered in honey bee larval food (bee bread) in Europe and the concentrations we chose to test in our experiment.

Pesticide	Type	Min (ng/g)	Max (ng/g)	Conc. used (ng/g)	References
Acetamiprid	Insecticide	<0.07	>100	100	(Beyer et al. 2018, Böhme et al. 2018, Daniele kail et al. 2018, Bokšová et al. 2021, Kaila et al. 2021)
Glyphosate	Herbicide	10	700	527.5	(El Agrebi et al. 2020, Bergero et al. 2021, Kaila et al. 2021, Raimets et al. 2020)
Thymol	Acaricide	146	481	146	(Manzano Sánchez et al. 2021)
Dimethoate	Positive chemical control	-	-	48000	(OECD 2016)

### Experimental setup

We assigned larvae to one group of each treatment type-*E. faecalis* dose and pesticide type and exposed them to their pesticide treatment throughout the experiment. We administered *E. faecalis* treatments in larval feed on day 1 of the experiment. Larvae exposed to high doses of *E. faecalis* received 4.266 × 10^6^ CFUs per ml and larvae exposed to low doses received 8.532 × 10^5^ CFUs per ml. Each pesticide treatment was administered to 120 larvae and each *E. faecalis* treatment was administered to at least 216 larvae with each unique treatment combination (pesticide and *E. faecalis* dose) administered to at least 36 larvae (details in Supplementary information).

**Figure 2.**
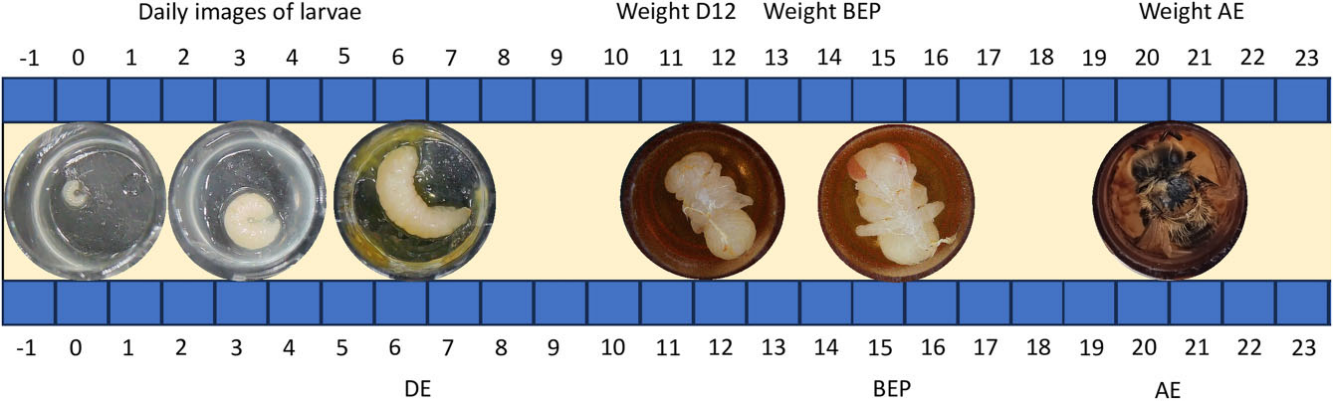
Measurements collected during larval development observed over 4 weeks. The day of Defecation events (DE), the appearance of brown-eyed pupa (BEP), and adult emergence (AE) were recorded. Daily images were taken to calculate larval growth. Weight was measured on day 12 (D12), when each individual became a brown-eyed pupa (BEP) and when they emerged as an adult bee (AE).

Each day, we monitored larval survival under a dissecting stereomicroscope (Fig. 2), with a lack of tracheal movements and no apparent response to physical stimuli indicating the death of individuals. We removed dead larvae from the tissue plate and cleaned the vacant cells with cotton buds and Sodium Carbonate following NBU sterilisation guidelines (National Bee Unit 2018). We provided 160μl of larval feed to each larva across their development. To prevent potential stress to larvae by repeated weighing, we estimated size using daily photos taken using a digital Andonstar (AD249S-M). This allowed us to record the 2D area (using ImageJ software) taken up by the larva in the cell (Schneider et al. 2012). This 2D metric is closely linked to insect larval mass (Smiley and Wisdom 1982) and has been used in various bee studies (Patel et al. 2007, Han et al. 2022, Kato et al. 2022).

### Measurements of pupae and adults

Once defecation occurred prior to pupation, we transferred the larvae to filter paper and then moved them to a clean tissue plate. If further defecation occurred on subsequent days, we cleaned the larvae again using tissue paper and repositioned them into the tissue plates cleaned using Sodium Carbonate and cotton buds. We monitored individuals daily, recording their defecation events (DE), becoming a brown-eyed pupa (BEP) and adult emergence (AE) using criteria defined by OECD rearing protocols (Oecd 2016). We weighed all individuals (wet weight) at the key milestones-BEP, AE, and on day 12 (D12).

### Analysis

To assess the survival of larvae depending on the *E. faecalis* and pesticide treatment they received, we used Cox proportional hazard models using the ‘survival’ package (Therneau 2024, RStudio Team 2024, details in Supporting Materials). Initially, we specified interactions between the two treatment types (*E. faecalis* dose and pesticide type); however, as there were no significant interactive effects, we defaulted to the use of a minimum adequate model removing the interactive effects when reporting the effects of *E. faecalis* and pesticide treatments on larval survivorship (Crawley 2007).

To assess the effects of the treatments on growth, we used linear mixed models (estimated using REML and *nloptwrap* optimiser) using the ‘lme4’ package to assess the sublethal effects of the treatments on the size of individuals and their rate of development, and hive of origin was fitted as a random variable (Kuznetsova et al. 2017, Therneau 2024, Bates et al. 2015). Subsequently, if model fit needed to be improved, stepwise backwards simplification was employed. Each of the models and their metrics are in the Supporting materials.

## Results

### Exposure to *E. faecalis* reduces brood survival and growth

**Figure 3.**
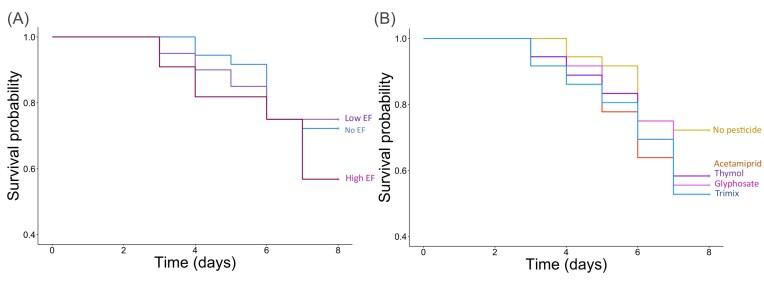
Independent effects of both treatment groups. Larval feed was spiked with *E. faecalis* on day 1 of the experiment, and pesticides were supplemented into feed throughout development. A) Effects of different doses of *E. faecalis* on larval survivorship over time. The survival rate over time in larvae unexposed to *E. faecalis* is shown in blue (*n* = 36); the survival rate of those exposed to low doses of E. faecalis (8.5 × 10^6^ CFUs per larvae) is shown in purple (*n* = 40); and the survival rate of those exposed to high doses of *E. faecalis* (4.2 × 10^7^ CFUs per larvae) is shown in red (*n* = 44). High doses of *E. faecalis* significantly reduced survival. B) Effects of pesticides on larval survivorship over time in larvae unexposed to *E. faecalis* (*n* = 36 per treatment). The treatments included a pesticide-free control in yellow; acetamiprid in orange; glyphosate in pink; thymol in purple and the trimix which contained acetamiprid; glyphosate and thymol in green. Acetamiprid, thymol, and trimix all significantly reduced larval survival.Treatments per line are also annotated on day 8 for clarity.

Overall, *E. faecalis* exposure reduced the survivorship of honey bee larvae (χ^2^ = 13.36, *P* = 0.0013, df = 2). High-dose treatments of *E. faecalis* significantly reduced survival to 56.8% compared to 72.2% in the *E. faecalis* free controls (HR = 1.38, 95% CI: 1.09–1.76, p = 0.008; Fig. 3A). The survivorship of low dose treated larvae was 75% which is similar to the *E. faecalis* free controls (HR = 0.94, 95% CI: 0.72–1.23, p = 0.64; Fig. 3A; Supporting tables).

**Figure 4.**
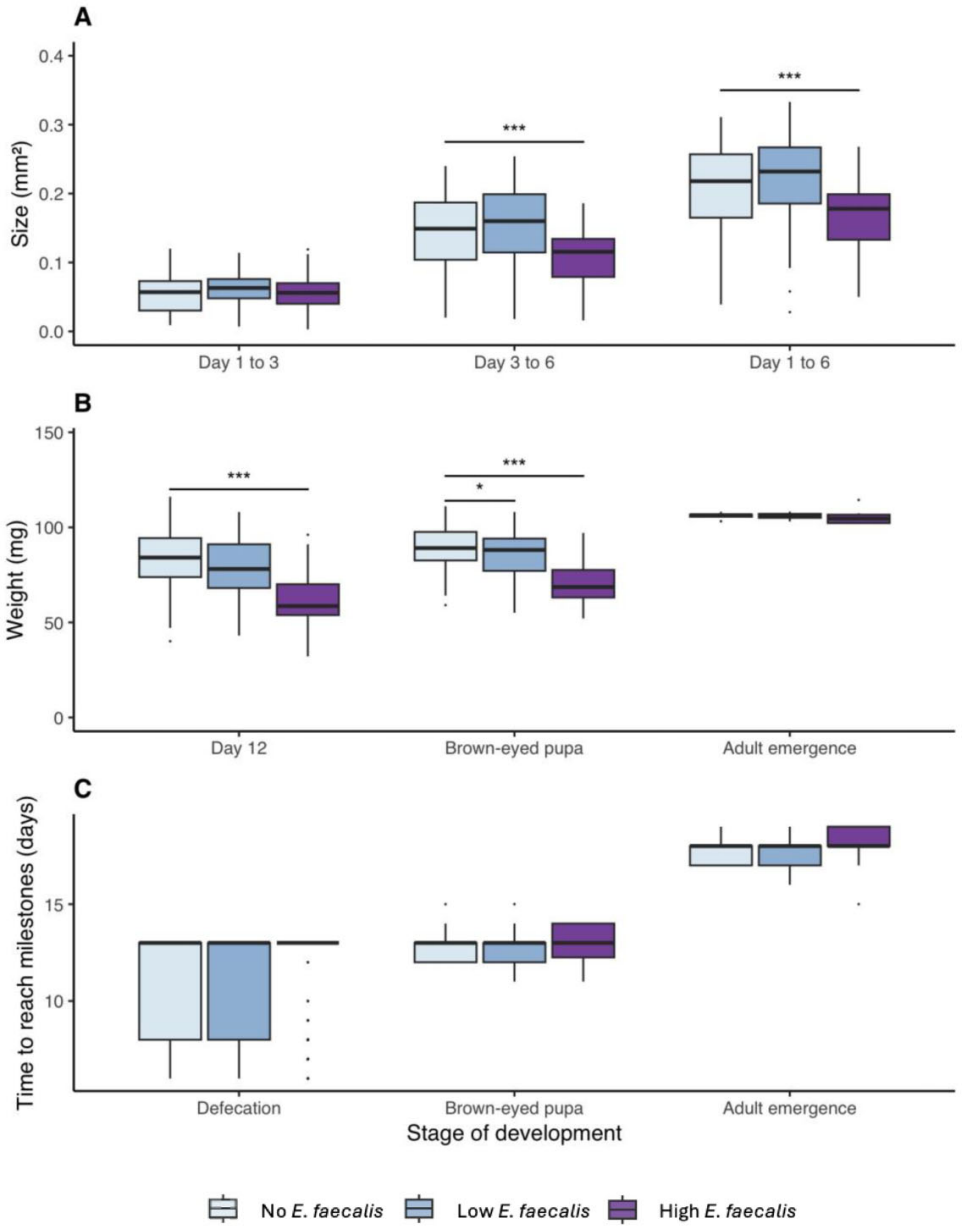
Sublethal effects of exposure to varying levels of *E. faecalis* on brood size and rate of development. A) Increase in area covered by larvae between day 1 and day 3 (*n* = 555), between day 3 and day 6 (*n* = 334) and between day 1 and day 6 (*n* = 334). B) Increase in mass in larvae measured upon death (*n* = 275), at day 12 of development (*n* = 241), when individuals become brown-eyed pupae (BEP) (*n* = 79), and when individuals emerge as adults (*n* = 66). C) Rate of reaching key stages of development depending on *E. faecalis* exposure, including defecation (*n* = 246), becoming a BEP (*n* = 79) and emerging as an adult (*n* = 66). Significant differences relative to controls have been indicated by asterisk * *P* <0.05, ** *P* <0.01,*** P <0.001.

By day 6, high doses of *E. faecalis* had significantly reduced larval growth by 22.5% relative to the growth observed in the control group (t_316_ = –3.35, 95% CI [–0.08, –0.02], p < 0.001). This reduction was most pronounced between day 3 and day 6, where the volume of larvae was 30.1% relative to controls (t_316_= −3.37, 95% CI [-0.01, 0.01], p < 0.001; Fig. 4A; Supporting tables). The smaller size of individual larvae exposed to high doses of *E. faecalis* persisted in post-larval stages. This was reflected in the reduction by 8.4% of pupal mass at day 12 (t_64_ = –4.34, 95% CI [–50.18, –18.52], p < 0.001), and 32.5% reduction in mass of brown-eyed pupae (t_225_ = –3.99, 95% CI [–27.56, –9.33], p < 0.001); Fig. 4B; Supporting tables). However, low doses of *E. faecalis* only reduced the mass of brown-eyed pupae by 19.8% (t_225_ = –0.74, 95% CI [–10.61, 4.80], p = 0.011). No life stages experienced significant changes in development speed during the *E. faecalis* treatments (Fig. 4B).

### Exposure to field-realistic concentrations of pesticides reduces honey bee brood survival and growth

Overall, pesticide treatment reduced larval survivorship (χ^2^ = 222.59, *P* < 0.001, df = 5; Fig. 3B). All pesticides, excluding glyphosate, significantly reduced survivorship in larvae. Compared to the overall survival in non-exposed larvae of 67.5%, acetamiprid lowered survival to 55.3% (HZ=1.61 (95% CI: 0.72–1.23), p = 0.023; Fig. 3B; Supporting tables), thymol to 50.0% (β = 0.58, *P* = 0.005; Fig. 3B), and the trimix to 49.2% (HZ=1.78 (95% CI: 1.19–2.66), p = 0.005; Fig. 3B). We found the positive control dimethoate to effectively reduce survival to 5% in larvae untreated with *E. faecalis* (HZ=1.78 (95% CI: 1.19–2.66), p = 0.005; Fig. 3B) and removed it from graphics.

**Figure 5.**
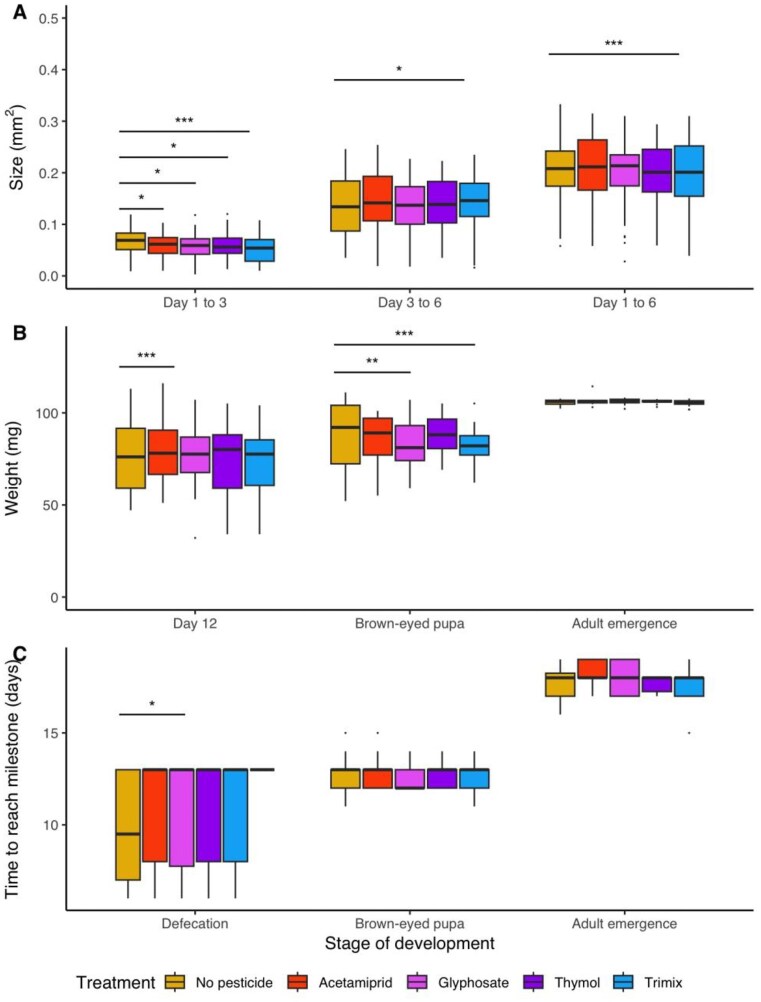
Sublethal effects of pesticide exposure on brood size and rate of development. A) Increase in area covered by larvae between day 1 and day 3 (*n* = 555), between day 3 and day 6 (*n* = 334) and between day 1 and day 6 (*n* = 334). B) Increase in weight at day 12 of development (*n* = 241), when individuals become brown-eyed pupae (*n* = 79), when individuals emerge as adults (*n* = 66). C) Rate of reaching key stages of development depending on pesticide exposure, including defecation (*n* = 246), becoming a brown-eyed pupa (*n* = 79) and emerging as an adult (*n* = 66). Significant differences relative to controls have been indicated by asterisk * *P* <0.05, ** *P* <0.01,*** P <0.001.

Pesticide exposure stunted larval growth over the first three days of development across all pesticide treatments relative to the unexposed controls (Fig. 5; Table 2). Larval size in younger larvae was lowered in the trimix treatment 30.8% (t_535_ = –3.57, (95% CI: –0.031, –0.009), p < 0.001; Fig. 5A)), by 23.0% in the glyphosate treatment (t_535_ = –2.76, (95% CI: –0.026, –0.004), p = 0.006; Fig. 5A), by 21.7% in the thymol treatment (t_535_ = –2.65, (95% CI: –0.027, –0.004), p = 0.008; Fig. 5A), and by 17.3% in the acetamiprid treatment (t_535_ = –2.02, (95% CI: –0.023, –0.0003, p = 0.044; Fig. 5A). In older larvae, growth was reduced by 30.1% in those exposed to the trimix (t_535_=-3.566, (95% CI: -1.30, -0.38), p<0.005; Fig. 5A); however, between day 3 to day 6, the trimix increased larval size change by 5% relative to control (t_316_ = -2.56, (95% CI: -0.067, -0.009), p = 0.011; Fig. 5A) indicating that the difference primarily stems from the earlier stages of larval development. A reduction in growth of individuals exposed to the trimix was also observed in brown-eyed pupae, where individuals were smaller by 4.7% than those in the control (t_64_ = –3.64, (95% CI: –38.99, –11.35), p < 0.001; Fig. 5A). Glyphosate also reduced the mass of brown-eyed pupae by 2.6% relative to the control (t_64_ = –3.00, (95% CI –34.99, –6.99), p = 0.004; Fig. 5B; Table 2). The time to reach developmental milestones was not impacted by pesticide exposure (Fig. 5C; Table 2)

**Table 2.** Summary of lethal and sublethal effects identified from the independent treatments with both treatment types-*E. faecalis* dose and pesticide type.

Treatment	Lethal effects	Sublethal effects								
	*Survival larva*	*Size of larva (mm^2^)*			*Weight of pupae and adults*			*Rate of development (days to milestones)*		
	*Day 1–6*	*Days 1–3*	*Days 3–6*	*Days 1–6*	*Day 12*	*Brown-eyed pupa*	*Adult*	*Defecation*	*Brown-eyed pupa*	*Adult*
Low dose *E. faecalis*	N.S.	N.S.	N.S.	N.S.	N.S.	↓	N.S.	N.S.	N.S.	N.S.
High dose *E. faecalis*	↓	N.S.	↓	↓	↓	↓	N.S.	N.S.	N.S.	N.S.
Acetamiprid	↓	↓	N.S.	N.S.	↓	N.S.	N.S.	N.S.	N.S.	N.S.
Glyphosate	↓	↓	N.S.	N.S.	N.S.	↓	N.S.	N.S.	N.S.	N.S.
Thymol	N.S.	↓	N.S.	N.S.	N.S.	N.S.	N.S.	N.S.	N.S.	N.S.
Trimix	↓	↓	↓	↓	N.S.	↓	N.S.	N.S.	N.S.	N.S.

↓ indicates a reduction in a metric as result of the corresponding treatment, and N.S. indicated no significant change.

### Combined chemical and *E. faecalis* exposure results had no interactive effects on survival but diverging effects on growth metrics

**Figure 6.**
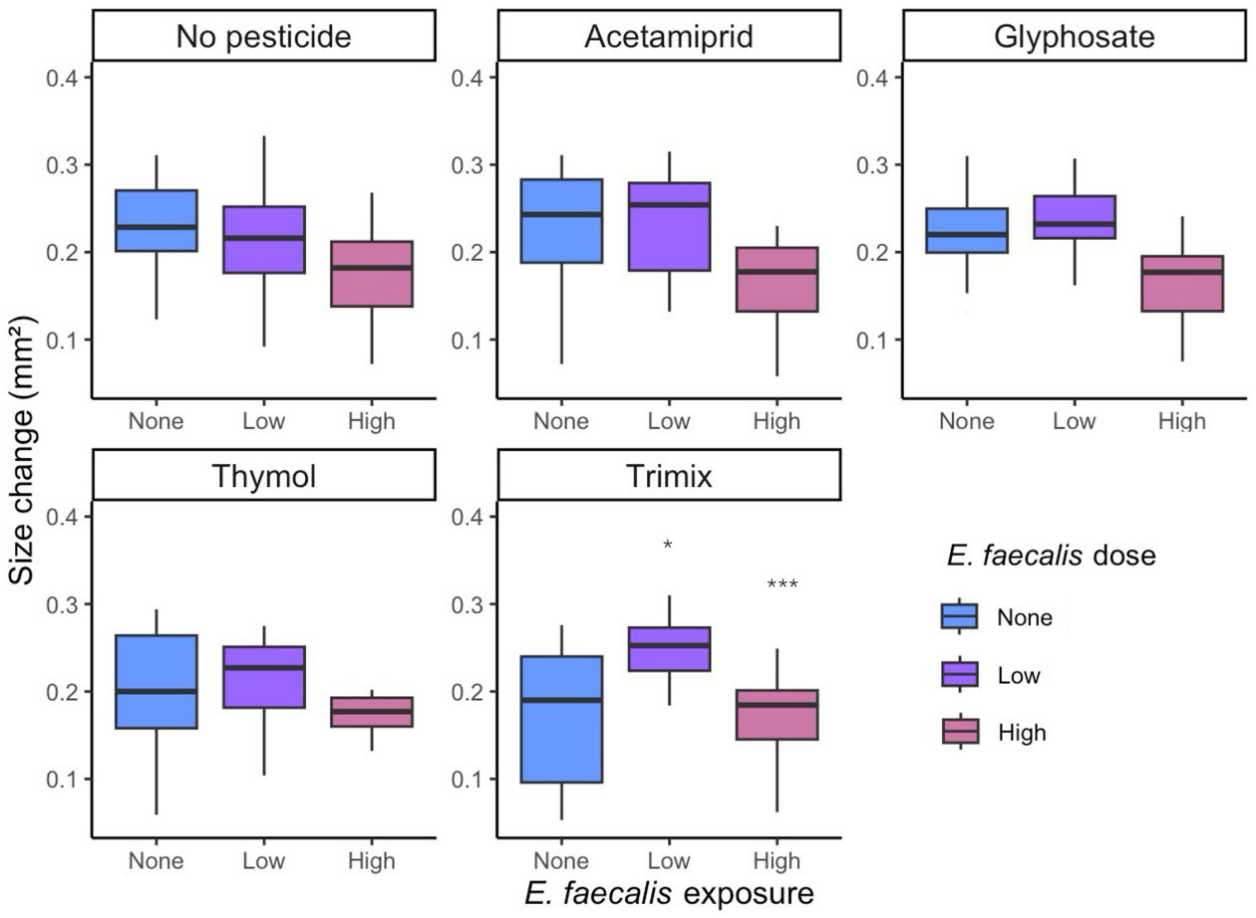
Effects of *E. faecalis* dose and pesticide treatment on the growth (mm^2^) of larvae as determined by the 2D area covered by larvae between day 1 and day 6 (*n* = 334). These were calculated from images using the image analysis software ImageJ (Schneider et al. 2012). Significant differences relative to controls have been indicated by asterisk * *P* <0.05, ** *P* <0.01,*** P <0.001.

Pesticide interactions with *E. faecalis* did not significantly affect larval survivorship (χ^2^ = 12.31, df = 10, *P* = 0.26; Supporting materials); however, larval size was impacted by the trimix’s interaction with *E. faecalis* (Supporting materials). Most exposure combinations did not result in an interactive effect on larval size, aside from the combination of high doses of *E. faecalis* and trimix, and low doses of *E. faecalis* and trimix. Co-exposure to the trimix and a low dose of *E. faecalis* increased larval size by 5.6% relative to the unexposed controls (day 1 to day 6 larvae, t_316_ = 4.34, (95% CI: 0.05, 0.14), p < 0.001; Fig. 6), with the later stages of larval development being 14.5% larger than controls (day 3 to day 6 larvae, t_315_ = 3.59, (95% CI: 0.033, 0.111), p < 0.001; Fig. 6). Larvae exposed to the same trimix of pesticides but a high dose of *E. faecalis* experienced the opposite effect and were 26.9% smaller than those unexposed to either treatment (day 1 to day 6, t_316_ = 2.10, (95% CI: 0.00, 0.10), p = 0.037; Fig. 6).

Most exposure combinations did not result in an interactive effect on the mass of individuals, aside from the combination of trimix with high or low doses of *E. faecalis*, and glyphosate with high or low doses of *E. faecalis*. Brown-eyed pupae exposed to the trimix of pesticides combined with low doses of *E. faecalis* weighed 12.9% more on average (t_64_ = 3.03, (95% CI: 9.42, 46.02), p = 0.004), while those treated with high *E. faecalis* and trimix were 32.1% smaller than controls (t_64_ = 2.09, (95% CI: 1.00, 44.34, p = 0.041). Similarly, the co-exposure to low-dose *E. faecalis* and glyphosate increased brown-eyed pupal mass by 16% (t(64) = 2.06, 95% CI [0.71, 44.28], p = 0.043), while high-dose *E. faecalis* and glyphosate also reduced brown-eyed pupae mass by 28.2% (t_64_ = 2.31, (95% CI: 2.85, 39.59), p = 0.024). The high dose of *E. faecalis* and glyphosate also deferred defecation by 17.1% relative to the control (t_229_ = 2.54, (95% CI: 0.28, 2.25), p = 0.012).

## Discussion

This is the first study to examine the independent and interactive effects of *E. faecalis* and pesticide exposure on the health of honey bee brood-an important consideration for hive health. We found that *E. faecalis* can impact honey bee larval survival and development indicating a risk to honey bee health if concentrations are high enough. When larvae were exposed to a mixture of different pesticide classes (trimix: the neonicotinoid acetamiprid, the herbicide glyphosate, and the acaricide thymol), we observed divergent effects on growth dependent on *E. faecalis* concentration. While high *E. faecalis* doses seem to reduce larval size and the mass of brown-eyed pupae, low doses have the opposite effect and result in larger larvae and brown-eyed pupae than those observed in the control group. This indicates that the dose of *E. faecailis* at field-realistic conditions can go from countering the negative effects of certain pesticides on larval growth, even being antagonistic, to exacerbating the pesticide effect, possibly even showing a synergistic effect. Similarly, larvae treated with glyphosate and high doses *of E. faecalis* grew into smaller brown-eyed pupae relative to the control, while those treated with glyphosate and low doses of *E. faecalis* grew to be larger than controls as brown-eyed pupae. Larvae treated with high doses of *E. faecalis* and glyphosate also experienced delayed defecation. Our results show how dependent host health is on the dosage of a commensal bacterium.

We found that high doses of *E. faecalis* reduced larval survival and the size of the developing brood (larvae and pupae). Like in several other systems, *E. faecalis* can be pathogenic in honey bee hosts (Mason et al. 2011, Guiton et al. 2013, Zahran et al. 2019). In the field, such impairment could reduce worker numbers, stifle colony growth, reduce pollination services and increase the likelihood of colony collapse. Whilst we do not identify the mode of pathogenicity in honey bees and did not assess whether *E. faecalis* had colonised the guts of larvae, in other insect systems, it has been shown to cause sepsis and death once within the haemocoel of its host, which may be facilitated by other pathogens that can degrade the gut lining and pave a way into the host haemocoel (Mason et al. 2011). This could indicate that a high prevalence of *E. faecalis* in hives infected with pathogens that breach the gut lining, such as *Melissococcus plutonius* and *Paenibacillus larvae*, are potentially at particularly high risk (Yue et al. 2008, Takamatsu et al. 2015). This could explain why it is a known secondary invader in European Foulbrood infections, exacerbating symptoms of the disease (Gaggìa 2015, Erban et al. 2017, Floyd et al. 2020, Sopko et al. 2020). Despite this, with no facilitator at low concentrations, *E. faecalis* might remain a benign commensal microbe, with the prospect of becoming an opportunistic pathogen.

At field-realistic conditions (trimix), low and high doses of *E. faecalis* had opposing effects on larval growth, with low doses resulting in increased size of larvae and brown-eyed pupae, and high doses resulting in reduced size relative to the control larvae. This could indicate that while high concentrations of *E. faecalis* may harm brood health, low concentrations may confer benefits. Another species from the genus, *E. faecium*, can upregulate developmental genes and support healthy brood development, which could also be the case for *E. faecalis* (Du et al. 2021). Furthermore, low doses of *E. faecalis* may confer other benefits to larval health through either improving their immunocompetence or enhancing their ability to utilise nutrients (Parish et al. 2022, De Beer et al. 2023, Ye et al. 2023). Other *Enterococcus* species have been shown to upregulate expression of AMP genes, which may improve their ability to cope with pathogenic stress (Ye et al. 2023). Future studies should aim to assess the value of the microbe to the host under pathogenic stress, as it might confer additional benefits at low doses. It is known that *E. faecalis* has antimicrobial effects against a range of pathogenic and spoilage-associated microbes, including the honey bee brood pathogen *P. larvae* and it also stimulate host immune responses against the pathogen, similarly to other lactic acid bacteria (Yoshiyama et al. 2013, Jaouani et al. 2014).

We did not find any pesticide-*E. faecalis* interactions that led to reduced survivorship in larvae; however, in adult honey bees, an interaction between *E. faecalis* and thiacloprid exposure has been found to improve survivorship in the short-term (Dickel et al. 2018). It has been proposed that such an interaction could be the product of hormetic effects mediated in a dose-dependent manner, as some pesticides may suppress the appetites of honey bees (Dickel et al. 2018). This would mean that when exposed to the microbe, some pesticides, at low concentrations, could promote improved survivorship; however, as the concentration increases, so do their negative effects on bee health. These short-term positive effects on survivorship, however, are unlikely to be sustained as poor nutrition as a result of suppressed appetite can also act as a stressor (Castelli et al. 2020). While all larvae were provided with the same amount of food throughout the study, individuals exposed to high *E. faecalis* concentrations and trimix consumed a lower volume of food. Appetitive behaviour may have played some role in this response, as glyphosate can reduce sucrose sensitivity and reduce reward-seeking behaviour (Herbert et al. 2014, Tan et al. 2022). This, however, does not explain why at low doses of *E. faecalis*, this effect was reversed, or why the glyphosate treatment combined with either *E. faecalis* dose did not reduce larval size and only reduced the mass of brown-eyed pupae. This indicates that while appetitive behaviours may play a role in mediating the interaction, there could be further factors at play.

The interaction between glyphosate and high *E. faecalis* concentrations reduced the mass of brown-eyed pupae. Transcriptome work has identified that glyphosate-exposed individuals experience changes in defence and metabolism (Vázquez et al. 2020). This is observable even in visually asymptomatic individuals, which may increase vulnerability to opportunistic pathogens (Vázquez et al. 2020). Another way the interaction could be mediated is through glyphosate disrupting the microbial community of its host, making them more vulnerable to exploitation by opportunistic pathogens (Dai et al. 2018, Motta et al. 2018). Glyphosate was the only pesticide out of the three we found to reduce pupal mass. Reports of sublethal effects or changes in immune system modulation with either thymol or acetamiprid have been minimal, even at higher concentrations than the field-realistic concentrations employed by our study (Glavan et al. 2020, Gao et al. 2022). It is therefore likely that the reduction in mass in the trimix group was primarily driven by the effects of glyphosate, where delayed development and reduced growth have both been reported for larvae exposed to this herbicide (Vázquez et al. 2018). This indicates that glyphosate can have harmful effects on larval development, which may either be counteracted at low doses of *E. faecalis*, or compounded by high doses of *E. faecalis*.

We identified divergent effects of different doses of *E. faecalis* when larvae were exposed to the trimix. This could be mediated by microbe-driven modulation of host immune responses or microbiome composition. Other commensals can also buffer the effects of chemical stressors in honey bees. For example, *Gilliamella apicola* contributes to the breakdown of toxic sugars such as mannose and rhamnose (Zheng et al. 2016, 2017), and its abundance increases under low-dose imidacloprid exposure, particularly in bees fed a natural pollen diet (Kakumanu et al. 2016). This microbial shift is associated with upregulation of pathways involved in xenobiotic degradation and carbohydrate metabolism, suggesting a potential compensatory or detoxifying role under pesticide stress. However, at high abundance, *G. apicola* can disrupt gut microbiome balance and contribute to dysbiosis, particularly when microbial communities are destabilized (Kwong et al. 2017, Kwong et al. 2017). This example suggests that other members of the honey bee microbiome may also have dual functions on the health of their host under pesticide exposure stress and highlights some of the mechanisms that may be applicable to the results we observe. We hope that future studies can examine the mechanism of the effects we observe by interrogating the effects *E. faecalis* has on the microbial communities of larvae and their immune function, as well as examine whether the consequences we observe in brood translate to adult bees and colony health overall.

## Supplementary Material

fiaf080_Supplemental_File
