# Supplementary material for: Friend or foe? Concentration of a commensal microbe induces distinct responses in developing honey bees exposed to field-realistic pesticide concentrations
Source: FEMS Microbiol Ecol. 2025 Jul 29;101(9):fiaf080. doi: 10.1093/femsec/fiaf080 (PMC12418955; doi:10.1093/femsec/fiaf080)
Supplement: fiaf080_Supplemental_File [file fiaf080_supplemental_file.docx]

## Supporting information

### Pesticide selection criteria

We determined the ranges at which our compounds of interest were encountered in bee bread through systematically searching *Web of Science* and *Google Scholar* using all permutations of the following search terms: "honey bee" or “honey bee” + "beebread" or “bee bread” + “pesticides” + "Europe". The field-realistic concentration of acetamiprid and glyphosate was selected to be approximately 75% of the maximum detected concentration. For glyphosate, the range at which it was detected in the literature was 10-700ng/g (1,2). Therefore, the food provisioned to larvae for the glyphosate treatment had a concentration of 527.5ng/g, for the acetamiprid was 0.65-171.4ng/g, and for acetamiprid was 128.713ng/g. As data for thymol was limited, we selected a field-realistic concentration of thymol based on a study examining its concentration across different matrices including beebread after a standard application in the colony. We selected the concentration measured the following year (146ng/g) after an autumn application as the colony is unlikely to be growing in winter and therefore is unlikely to be affected by the higher concentration (481ng/g) of the compound even if any effects are found to be associated with it (3). Pesticides accumulate in beebread and other matrices causing the exposure of larvae to cocktails of chemicals (4). Therefore, each of the three selected pesticides was applied both independently and in combination with the other two (trimix). Additionally, we utilised dimethoate as a positive control for our chemical treatments at the standard concentrations 48mg/Kg (5).

### Preliminary trial: Optimising larval feed

We aimed to select a single diet formulation that can support larval health throughout development as formulations for younger larvae tended to be less concentrated in sugars and yeast than those for older larvae. Therefore, we reviewed three standard methods papers, each containing three diet formulations and selected two of those formulations which had middle ranges of glucose, fructose and yeast (5–7). The two diets selected were OECD Diet A and Schmehl Diet C (5,7). We reared larvae according to protocols outlined in our paper, rearing 96 larvae on each of the two selected diets. The larvae in each treatment group had equal proportions of larvae from three different hives obtained at Silwood park. We found that survival of larvae reared on Schmehel Diet C had higher survivorship relative to those reared with diet OECD-A (Supporting figure 1) and therefore used this diet throughout our experiment.


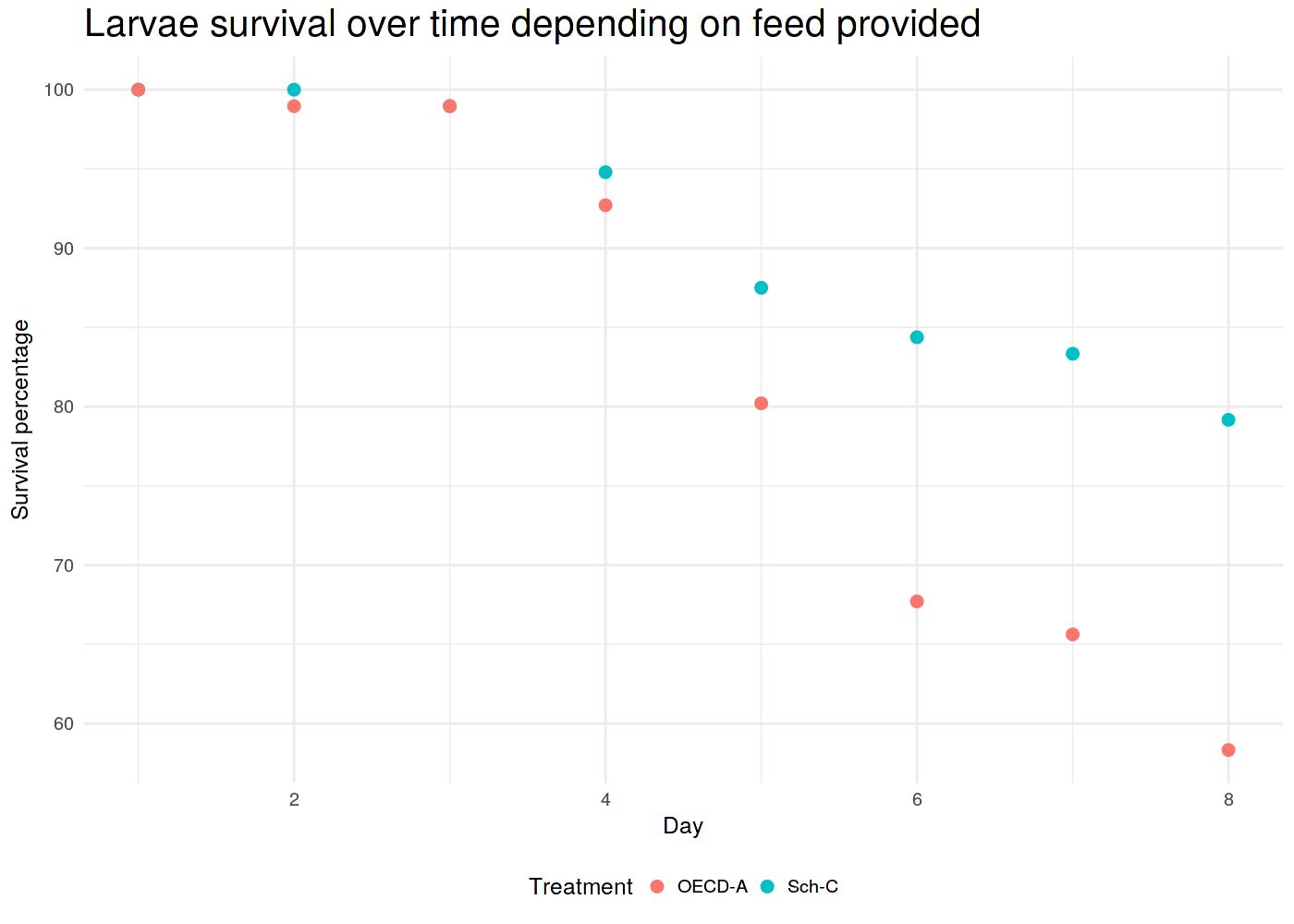


*Supporting figure 1: Survival of larvae exposed to 2 diet regimens selected from standard methods papers at different days of development (n=192).*

### Predicting the number of *E. faecalis* CFUs to be administered to larvae to achieve desired growth

To deduce the number of introduced CFUs, we suspended one CFU of *E. faecalis* in liquid media and prepared serial dilutions plated onto solid media. We then counted the number of CFUs resulting on the plates and took the dilution factors into account to estimate the number of CFUs per μl in the original solution. To predict this prior to the experiment, we had previously repeatedly performed this procedure, to enable us to anticipate the number of CFUs we would be introducing to the larvae, however as the size of CFUs varied, the outcomes of this were also variable. Therefore, we determined the relationship between CFU growth and OD in liquid media to measure the OD prior to administering *E. faecalis* into the feed and determine the appropriate amount of solution that would need to be administered to produce the desired amount of CFUs.

To determine the relationship between OD and CFU growth, we set up 96-well tissue plates (scheme in Supporting figure 2). We set water margins with 180μl of water in each of the wells around the edges of the plate and used 180μl of BA media in our control wells. In treatment wells, we administered 175μl of BA media and 5ul of liquid BA media containing 1 of 3 pre-prepared *E. faecalis* CFUs. Every 3 hours, we measured the ODs from the plate and created serial dilutions from 1 experimental well for all 3 of the CFUs. The resultant serial dilutions were plated on solid BA media to determine the number of CFUs that result from the measured OD. Taking into account dilutions, we were able to determine the relationship between CFUs produced and OD by creating a linear regression equation with a known slope and intercept. By preparing a new 96-well plate in the morning after grafting honey bee larvae and measuring the OD at least 3h after the preparation of plate, we were able to determine the number of CFUs in the original raw culture of BA media containing a fresh *E. faecalis* CFU.

To prepare the feed for each of the treatments containing 1000μl of larval feed, we extracted 10μl of feed and replaced it with 10μl of liquid depending on their treatment. To prepare the feed for the larvae exposed to high concentrations of *E. faecalis*, we displaced the 10μl of feed with 10μl the raw *E. faecalis* culture suspended in liquid BA media. To prepare the feed for the low dose, we diluted the raw culture by a factor of 5 and displaced 10μl of the feed with this new diluted culture. For the control treatment, we displaced 10μl of larval feed with distilled water.


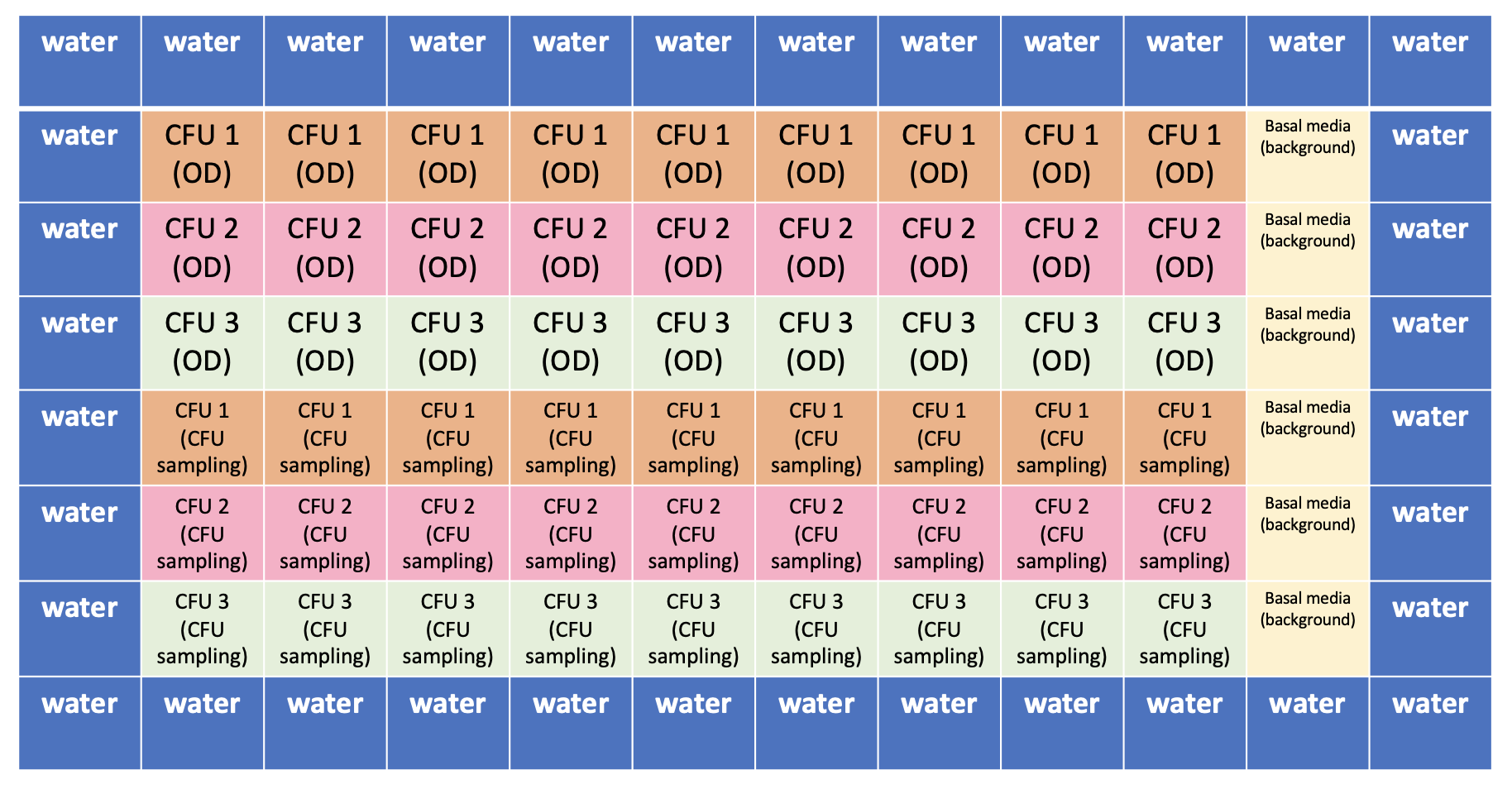


*Supporting figure 2: Layout of tissue plates used to determine the relationship between OD and CFUs produced from E. faecalis at different points of growth.*

### Treatments

*Supporting table 1: Number of larvae initially exposed to each treatment combination*

| Pesticide treatments | *E. faecalis* treatments | Number of larvae |
| --- | --- | --- |
| No pesticide | No *E. faecalis* | 36 |
| No pesticide | Low *E. faecalis* | 40 |
| No pesticide | High *E. faecalis* | 44 |
| ACE | No *E. faecalis* | 36 |
| ACE | Low *E. faecalis* | 40 |
| ACE | High *E. faecalis* | 44 |
| GLY | No *E. faecalis* | 36 |
| GLY | Low *E. faecalis* | 40 |
| GLY | High *E. faecalis* | 44 |
| THY | No *E. faecalis* | 36 |
| THY | Low *E. faecalis* | 40 |
| THY | High *E. faecalis* | 44 |
| TRI | No *E. faecalis* | 36 |
| TRI | Low *E. faecalis* | 40 |
| TRI | High *E. faecalis* | 44 |
| DIM | No *E. faecalis* | 36 |
| DIM | Low *E. faecalis* | 40 |
| DIM | High *E. faecalis* | 44 |

### Survival models

Model integrating interactions between treatments: coxph(Surv(dod, death) ~ EF + pesticides + (1|hive) + EF * pesticides, data = EF_pesticides)

The model compares each treatment to the non-exposed control larvae (Supporting table 2).

*Supporting table 2: Outcomes from survival model integrating interactions*

*between treatments.*

| **EF** | **pesticide** | **estimate** | **standard error** | **p** |
| --- | --- | --- | --- | --- |
| low | none | -0.08276 | 0.44722 | 0.8532 |
| high | none | 0.5338 | 0.39069 | 0.1718 |
| none | ACE | 0.67643 | 0.39855 | 0.0897 |
| low | ACE | -0.15302 | 0.57053 | 0.7885 |
| high | ACE | -0.35372 | 0.50312 | 0.482 |
| none | GLY | 0.54458 | 0.40313 | 0.1767 |
| low | GLY | -0.22667 | 0.57787 | 0.6949 |
| high | GLY | -0.30751 | 0.50677 | 0.544 |
| none | THY | 0.4956 | 0.40826 | 0.2248 |
| low | THY | -0.1446 | 0.58617 | 0.8052 |
| high | THY | 0.25359 | 0.50066 | 0.6125 |
| none | TRI | 0.65049 | 0.39854 | 0.1026 |
| low | TRI | -0.08625 | 0.56686 | 0.8791 |
| high | TRI | -0.15226 | 0.49717 | 0.7594 |

Model used: coxph(Surv(dod, death) ~ EF + pesticides + (1|hive), data = EF_pesticides)

This model was selected as the minimum adequate model (outcomes in Supporting table 3) relative to the more complex model above. The complex model had not identified any interactive effects (Supporting table 2) and had a worse AIC score (AICM1= 4606.781, AICM2= 4599.093).

*Supporting table 3: Outcomes from survival model*

| **EF** | **pesticide** | **estimate** | **standard error** | **p** |
| --- | --- | --- | --- | --- |
| low | CTRL | -0.06306 | 0.13592 | 0.64267 |
| high | CTRL | 0.32528 | 0.12296 | 0.00816 |
| none | ACE | 0.47381 | 0.20859 | 0.02312 |
| none | GLY | 0.341 | 0.21019 | 0.10473 |
| none | THY | 0.57618 | 0.2058 | 0.00512 |
| none | TRI | 0.55481 | 0.20506 | 0.00682 |

### LMM model construction for sublethal effects

We reported the results of each of the models assessing sublethal effects in *Supporting table 4, Supporting table 5, Supporting table 6* and *Supporting table 7.*

*The effects of E. faecalis and pesticide exposure on the increase in size*

As outlined in the larval rearing section, we used images and image processing software to determine the area taken up by each larvae within their cell daily. We used this data to create three LMMs to assess the effects of *E. faecalis* and pesticide exposure on the increase in size incurred at different stages of larval development – between day 1 and 3 (D1-D3), between day 3 and 6 (D3-D6) and overall, between day 1 and 6 (D1-D6). In all these models, we integrated interactive effects between *E. faecalis* dose and pesticides and fitted hive of origin as random effect.

*The effects of E. faecalis and pesticide exposure on the increase in weight*

In addition to size (mm^2^), we also measured the weight of individuals at critical developmental points – On day 12 of development (D12), when they developed brown-eyes, and upon eclosion as adults. For weight of individuals, we constructed LMMs which incorporated the interaction between *E. faecalis* dose and pesticides and fitted colony as random effect.

*The effects of E. faecalis and pesticide exposure on rate of reaching developmental milestones*

We reported the day of DE, becoming a BEP and AE (*Supporting table 4; Supporting table 7)*. We constructed three LMMs to assess the effects of *E. faecalis* dose and pesticides on the rate of reaching these key stages. In all models, we incorporated interactive effects between our treatments. We also included hive of origin as a random effect in two of the three models, not including in the model assessing rate of becoming brown-eyed pupae as the random effect was too small.

*Supporting table 4: Model metrics for models assessing sublethal metrics including size (area taken up) of larvae, weight during post-larval development and days taken to reach key life stages.*

*
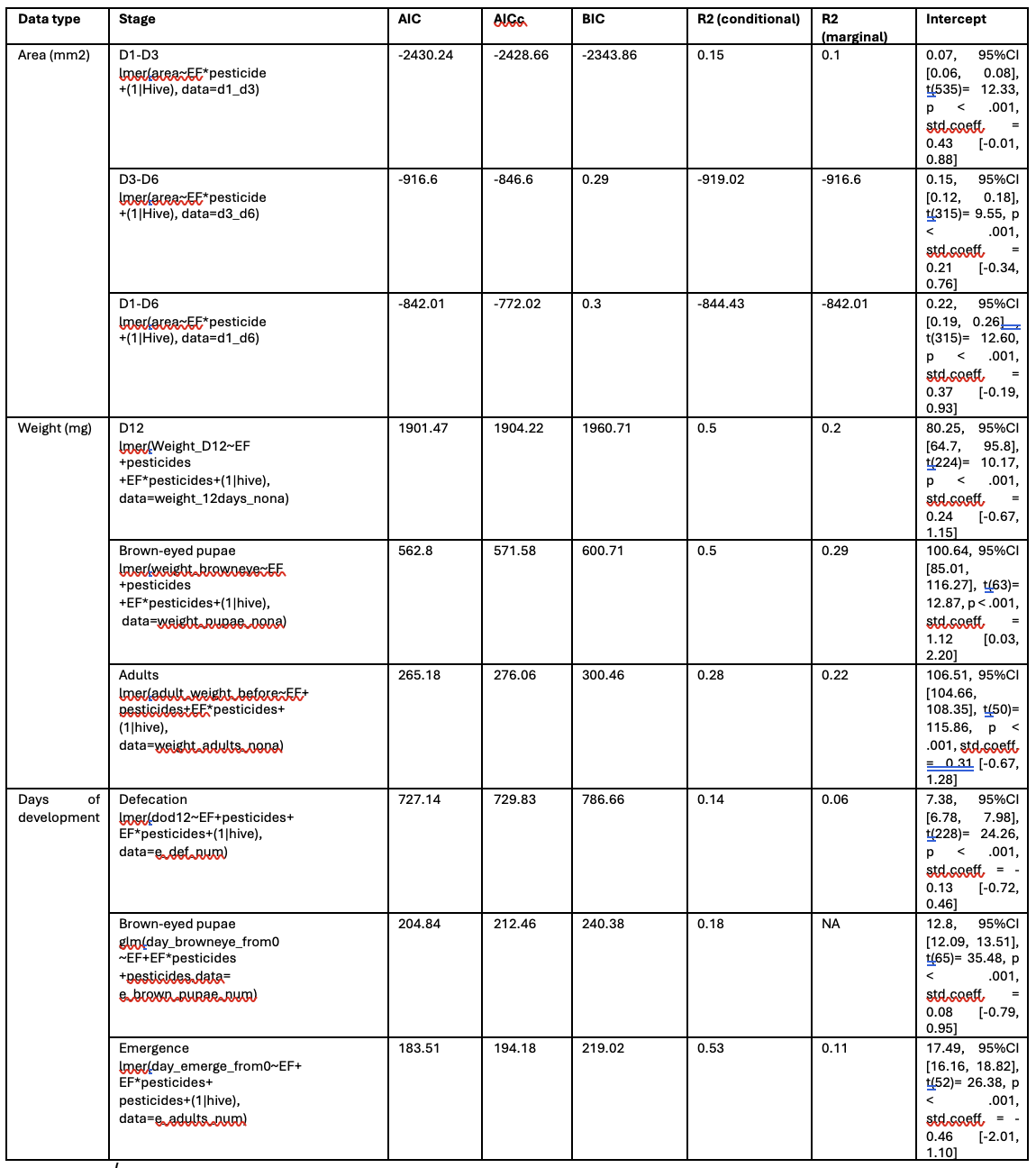
*

*Supporting table 5: Results for models assessing the effects of E. faecalis and pesticides on size (area taken up) of larvae.*

**

*Supporting table 6: Results for models assessing the effects of E. faecalis and pesticides on weight of individuals.*

**

*Supporting table 7: Results for models assessing the effects of E. faecalis and pesticides on rate of development.*

*Supporting figure 3: Relationship between E. faecalis dose and days of survival of larvae (n=720).*

## References for Supporting information

1. El Agrebi N, Tosi S, Wilmart O, Scippo ML, de Graaf DC, Saegerman C. Honeybee and consumer’s exposure and risk characterisation to glyphosate-based herbicide (GBH) and its degradation product (AMPA): Residues in beebread, wax, and honey. Sci Total Environ [Internet]. 2020;704. Available from: http://dx.doi.org/10.1016/j.scitotenv.2019.135312

2. Rutkowski D, Litsey E, Maalouf I, Vannette RL. Bee-associated fungi mediate effects of fungicides on bumble bees [Internet]. bioRxiv. 2021 [cited 2024 Jun 15]. p. 2021.09.06.459164. Available from: https://www.biorxiv.org/content/10.1101/2021.09.06.459164v1

3. Manzano Sánchez L, Gómez Ramos MJ, Gómez-Ramos M del M, Parrilla Vazquez P, Flores JM, R․ Fernández-Alba A. Presence, persistence and distribution of thymol in honeybees and beehive compartments by high resolution mass spectrometry. Environmental Advances [Internet]. 2021 Oct 1;5:100085. Available from: https://www.sciencedirect.com/science/article/pii/S2666765721000569

4. Gill RJ, Ramos-Rodriguez O, Raine NE. Combined pesticide exposure severely affects individual-and colony-level traits in bees. Nature [Internet]. 2012 Nov 1;491(7422):105–8. Available from: http://dx.doi.org/10.1038/nature11585

5. Oecd. Guidance document on honey bee (Apis mellifera) larval toxicity test, following repeated exposures. OECD Environment, Health and Safety Publications Series on Testing and Assessment [Internet]. 2016;34(207):1–16. Available from: http://www.oecd.org/officialdocuments/displaydocumentpdf?cote=env/jm/mono(2010)46&doclanguage=en

6. Crailsheim K, Brodschneider R, Aupinel P, Behrens D, Genersch E, Vollmann J, et al. Standard methods for artificial rearing of Apis mellifera larvae [Internet]. Vol. 52, Journal of Apicultural Research. 2013. Available from: http://dx.doi.org/10.3896/IBRA.1.52.1.05

7. Schmehl DR, Tomé HVV, Mortensen AN, Martins GF, Ellis JD. Protocol for the in vitro rearing of honey bee (Apis mellifera L.) workers. J Apic Res [Internet]. 2016 Sep 12;55(2):113–29. Available from: http://dx.doi.org/10.1080/00218839.2016.1203530
